# Research on the spatial association network structure for innovation efficiency of China’s new energy vehicle industry and its influencing factors

**DOI:** 10.1371/journal.pone.0255516

**Published:** 2021-08-26

**Authors:** Yining Zhang, Zhong Wu

**Affiliations:** Business School, University of Shanghai for Science and Technology, Shanghai, China; China University of Mining and Technology, CHINA

## Abstract

It is of great significance to study the spatial network of the new energy vehicle (NEV) industry innovation efficiency and its factors to promote the rational allocation of innovative resources and the coordinated development of Chinese NEV industry. First, the Super Efficiency Data Envelope Analysis model is used to measure innovation efficiency in the NEV industry in Chinese provinces, and based on the results, the improved gravity model is applied to construct a spatial correlation network. Then, by applying social network analysis (SNA) to study NEV industry development node spatial correlations, we conclude that there is no overall hierarchical structure. The SNA are applied to examine spatial correlations with respect to NEV industry innovation efficiency in each province, and to analyze the role and position of each province in the spatial correlation network. Finally, the influencing factors of spatial correlation of the innovation efficiency of China’s NEV industry has been discussed. The result shows that the difference in spatial distance and R&D investment has a significant impact on the spatial correlation of the NEV industry.

## Introduction

The increasing number of traditional cars has exacerbated environmental pollution and the energy crisis, and in order to address these issues, the NEV have attracted people’s attention for their environmentally friendly performance [1,2]. Due to the extent of science and technology application to automobile development, the NEV are becoming more and more popular. The NEV not only protect the environment, but also promote rapid development of related industries, including those associated with NEV fuel cells and charging systems, which will in turn support rapid expansion of national and regional economies. Countries in the world promote the NEV industry development in various ways, such as by issuing free license plates, awarding government subsidies, constructing more charging points, and offering tax incentives [3]. There is the largest world automobile consumer market in China, but while cars bring convenience to the society, they can also have a negative impact on air quality and human health. Hence, The Chinese government has committed to addressing these issues through NEV development. The central government in China has issued various policies to support development of the NEV industry in recently years, including “The development plan for energy saving and the NEV industry,” issued by the central government in July 2012. In September 2015, with Premier Li Keqiang presiding over a State Council meeting, a policy to increase support for the NEV industry, including associated R&D activities, such as power cells and fuel cell vehicles, was approved. The government’s support for the NEV industry has expanded the scale of the NEV industry, and has also increased the frequency of technical personnel, resources and other factors flowing between different regions. With the promotion of regional coordinated development strategies, the NEV industry has gradually formed a complex network structure. But the coordinated development of the NEV industry is restricted by traditional policies. According to current research [4,5], the development of the NEV industry presents regional imbalances in China. China’s NEV industry is mainly concentrated in the central and eastern coastal provinces such as Guangdong, Jiangsu and Anhui. Compared with underdeveloped regions, these regions have greater competitive advantages in terms of human capital, openness, investment intensity. The imbalance of regional development inhibits the coordinated development of China’s NEV industry. To advance the coordinated development of NEV industry, we should grasp the spatial correlation network of the development of China’s NEV industry from the overall perspective, explore the factors that affect the spatial correlation of the NEV industry, and reveal the formation mechanism of the spatial relationship, which is conducive to promoting the coordinated development of China’s NEV industry. In this context, this article analyzes the network structure and the changing trend of innovation efficiency in the spatial correlation network of the NEV industry. It also studies the role of each region in the spatial correlation network of the innovation efficiency of the NEV industry, and further explores the impact factors of the spatial correlation of innovation efficiency.

The research on new energy vehicle innovation is mainly from two levels of enterprise and region. From the perspective of enterprise research, Liu et al. took the NEV enterprise of Toyota, Tesla and BYD as the examples, they analyzed the ecological environment innovation, market positioning, innovation path and business model of these NEV companies [6]. Based on the SBM model and the network DEA model, Chen et al. constructed an evaluation index system for the technological innovation efficiency of Chinese NEV companies. They measured the technological innovation efficiency of China’s NEV enterprises from two aspects of technology research and development stage and achievement transformation stage [7]. Fang et al. combined Tobit regression with data envelopment analysis (DEA) to analyze the innovation efficiency of 23 new energy vehicle listed companies from 2013 to 2018 [8]. With using the improved general combined-oriented CCR model, Li et al. conducted empirical assessment of technical innovation efficiency, pure technical innovation and scale efficiency for China’s major car producers [9]. Wu et al. studied how the technological capabilities (measured by R&D expenditure) of NEV companies affect their eco-innovation performance (measured by eco-innovation patents) [10]. From the perspective of regional research, Xu et al. used the stochastic frontier model to analyze the innovation efficiency of China’s NEVs and its influencing factors. The results showed that the average value of innovation efficiency in the NEV industry is generally low. Compared with other regions, the Yangtze River Delta and the Pearl River Delta have the highest innovation efficiency [11]. Gao et al. used principal component analysis and DEA model to evaluate the innovation efficiency of NEV industry in 11 cities in 2014 [12].

In summary, the existing literature has extensively discussed the NEV industry. However, these documents mainly focus on efficiency measurement and efficiency evaluation. Due to the large regional differences in the development of the NEVs, especially in the context of the integration of the Chinese market, production factors such as capital, talents, and technology have formed cross-regional liquidity [13], and the innovation efficiency shows the characteristics of network relevance, modularity, and sub-group cohesion. Researching on the NEV industry from the perspective of individual effects alone, it cannot objectively reflect the development status of the NEV industry. Therefore, this article studies the innovation efficiency of the NEV industry from the perspective of spatial correlation. It can more objectively reflect the development characteristics of the NEV industry and provide policy recommendations for promoting the coordinated development of China’s NEV industry. Based on the existing literature, this article has expanded in the following aspects.

1. This article investigates the spatial correlation of the innovation efficiency of the NEV industry from a larger spatial scope. The current literature studies the NEV industry from the perspective of regional innovation efficiency differences, and regards the regional innovation system as an independent entity.
2. Compared with traditional spatial measurement methods, social network analysis (SNA) breaks through the limitations of traditional "attribute" data analysis, and can check relationship data and network relationships.
3. The article uses social network analysis methods to reveal the network structure characteristics and influencing factors of the innovation efficiency of the inter-provincial NEV industry, and provide policy recommendations for the NEV industry to achieve regional synergistic improvement.

## Methods and data

In order to study the spatial correlation of innovation efficiency of the NEV industry, this article conducted the following research: First, the paper uses the super-efficiency DEA to measure the regional innovation efficiency of China’s NEV industry. Second, we obtain the spatial correlation matrix of the innovation efficiency of the NEV industry through the improved gravity model. Third, we use the social network analysis method to determine the spatial correlation of the innovation efficiency of the NEV industry Matrix analysis.

### 2.1 Super-efficiency DEA model

The super-efficiency DEA model is an improved version of the traditional DEA models, such as the C2R decision-making model, in which the scale and technical effectiveness of decision-making units (DMUs) are evaluated simultaneously. The BC2 model has also been used specifically for evaluating DMU technical effectiveness, however, neither the C2R nor the BC2 model has been able to compare and sort DMUs. In contrast, the super-efficiency DEA model could compare and sort DMUs, resolving these traditional model shortcomings, and by applying this improved version, relative DMU effectiveness could be determined. The calculated efficiency value was no longer limited to the range of 0–1, as the improved model allowed values to exceed 1, as well as comparing and sorting DMUs. To describe the super-efficiency DEA model, it should first be assumed that there were *N* DMUs, and that their input and output data are respectively (*X*_*j*_, *Y*_*j*_*)*, *(j = 1*, *2*, *…*, *N*). The super-efficiency value evaluation equation for the j0 DMU of the super-efficiency DEA model can then be derived as shown in Eq (1):

$$\{\begin{array}{c} \min\theta -\varepsilon (\sum_{i=1}^{m}S_{i}^{-}+\sum_{i=1}^{m}S_{i}^{-})\\ \sum_{j=1}^{n}X_{ij}\lambda_{j}+S^{-}=\theta_{xj0},j=1,2\cdots ,m\\ \sum_{j=1}^{n}X_{ij}\lambda_{j}+S^{+}=\theta_{xj0},j=1,2\cdots ,m\\ \lambda_{j},S_{i}^{-},\geq 0,j=1,2\cdots ,j-1,j+1,n \end{array}$$ (1)

*θ* represents the super efficiency value of the *j*_*0*_^*th*^ DMU, and *ε* indicates a non-Archimedean infinitesimal. *N* shows the number of DMUs, and each DMU includes *M* input variables and *S* output variables. *S*_*i*_^*-*^ and *S*_*r*_^*+*^ are the input and output relaxation variables respectively. *X*_*ij*_ indicates that the *j*^*th*^ DMU is on the *i*^*th*^ input (input) index value, while *Y*_*rj*_ represents the value of the *j*^*th*^ DMU on the *R* output index value. *λ*_*j*_ represents the weight coefficient of the input and output indexes, while *θ*_*j*_, *λ*, *Y*_*rj*_, *S*_*i*_^*-*^, and *S*_*r*_^*+*^ are unknown values which can be solved by the model.

When *θ* ≥ 1, and *S*_*i*_^*-*^ = *S*_*r*_^*+*^ = 0, the *J*_*0*_^*th*^ DMU is said to be both DEA efficient, and scale and technology efficient. The larger the value of *θ*, the stronger its effectiveness.

When *θ* ≥ 1, and *S*_*i*_^*-*^ ≠ 0, or *S*_*r*_^*+*^ ≠ 0, then the *J*_*0*_^*th*^ DMU is referred to as being weakly DEA efficient, while when *θ* < 1, or *S*_*i*_^*-*^ ≠ 0, and *S*_*r*_^*+*^ ≠ 0, the *J*_*0*_^*th*^ DMU is referred to as being DEA invalid, meaning that it is either scalar invalid or technologically invalid.

### 2.2 The modified gravity model

Since the social network analysis method is based on "relational data", the determination of the spatial correlation of innovation efficiency between regions is the key to research. At present, the Granger causality test and the gravity model are generally used to describe spatial associations [14,15], with the gravity model chosen in this research to study spatial correlations. Compared with the vector autoregression (VAR) model, the gravity model is not only more suitable for total data, but it also takes economic and geographic distance into account quite comprehensively, before using cross-sectional data to describe SAN evolution trends [16]. We therefore selected this model to characterize evolutionary trends in the Chinese NEV industry innovation efficiency spatial correlation network. The modified gravity model is shown in Eq (2):

$$F_{ij}=k_{ij}\frac{\sqrt{P_{i}E_{i}}*\sqrt{P_{j}E_{j}}}{D^{2}{}_{ij}}\;k_{ij}=\frac{E_{i}}{E_{i}+E_{j}},$$ (2)

*i* and *j* represent provinces, and *F*_*ij*_ indicates the strength of the NEV industry innovation efficiency correlation between provinces *i* and *j*. *E*_*i*_ and *E*_*j*_ represent the NEV innovation efficiency in provinces *i* and *j* respectively, while *P*_*i*_ and *P*_*j*_ indicate the industrial enterprise R&D personnel in provinces *i* and *j*. Metric *D*_*ij*_ represents the distance between the two provincial capitals.

Eq (1) allowed the gravity matrix representing the extent of NEV industry innovation efficiency correlation between provinces to be calculated. In our study, gravitation values in each matrix row that are above average are denoted as 1, showing that there is a correlation between that province and other provinces. If the value is below average, it is denoted as 0, indicating that there was no such NEV industry innovation efficiency correlation.

### 2.3 Social network analysis

Compared with traditional spatial measurement methods, Social network analysis (SNA) breaks through the limitations of traditional "attribute" data analysis [17], and the SNA method has been used in spatial correlation network analysis of agriculture [18], energy [19], and environment [20–22] in recent years. As the cross-regional flow of innovation elements is increasingly becoming a trend, the spatial association of regional innovations presents a multi-threaded and complex networked feature, so the paper uses the SNA to analyze the overall network characteristics, individual network characteristics, plate model, and middlemen’s role of the spatial correlation network of China’s NEV industry innovation efficiency.

#### (1) Indicators of the overall network

Network density, network relevance, network level and network efficiency are used to characterize the overall network structure.

Network density is an indicator used to measure the degree of closeness between individuals in the network. The larger of the network density value indicates the closer of the spatial correlation degree of the innovation efficiency of the NEV industry. The calculation formula of network density is as:

D=n/[N(N−1)] (3)

*D* represents network density. *n* represents the actual number of relationships in the entire network. *N* represents the number of nodes in the entire network.

The degree of network relevance indicates the degree of reach between points in the network, and reflects the robustness of the network structure. The calculation formula for network relevance is shown as:

C=1−V/[N(N−1)/2] (4)

*C* represents the degree of network relevance. *V* represents the number of unreachable member pairs in the network. *N* represents the size of the network.

The network hierarchy expresses the degree to which the members in the network are asymmetrically reachable, and it reflects the hierarchical structure and dominance of each member in the network. The calculation formula of the network level is as:

H=1−M/max(M) (5)

*H* represents the level of the network. *M* represents the number of symmetrically reachable member pairs in the network.*max (M)* represents the largest possible number of symmetrically reachable member pairs.

Network efficiency is an indicator used to measure the degree of redundant lines in the network. The calculation formula of network efficiency is shown as:

E=1−K/max(K) (6)

*E* represents network efficiency. *K* represents the number of redundant lines.*max (K)* represents the maximum possible number of redundant lines.

#### (2) Indicators of the individual network

The structural characteristics of points in a network can be described by point, betweenness and closeness.

The point centrality indicates the number of other actors directly connected to the actor, and is used to measure whether each region is in the core position of the independent innovation efficiency correlation network. The calculation formula for relative degree centrality is shown as:

$$C_{\mathrm{RD}}=n/(N-1)$$ (7)

*n* represents the number of individuals directly associated with a point, and *N* represents the number of individuals in a network.

The closeness centrality reflects the degree to which the node is not controlled by other nodes. The greater the value close to the centrality, the closer the relationship between the node and other nodes. then the closeness of point *i* is shown as:

$$C_{AP}{}_{i}=\frac{n-1}{\sum_{j=1}^{N}d_{ij}}*100$$ (8)

*d*_*ij*_ represents the shortcut distance between regions *i* and *j*. *N* represents the number of regions in the network.

Betweenness centrality is an indicator reflecting the role of a point serving as a “bridge” and is measured with the help of the number of shortcuts. the betweenness of point *i* is as follows:

$$C_{ABi}=\frac{\sum_{j}^{n}\sum_{k}^{n}b_{jk}(i)}{n^{2}‐3n+2},j\neq k\neq i,and\;J<K$$ (9)

*g*_*jk*_ is the number of the shortcuts between provinces *j* and *k*; and *g*_*jk*_*(i)* is the number of shortcuts crossing province *i*, located between province *j* and province *k*. Let *b*_*jk*_*(i)* = *g*_*jk*_*(i)*/*g*_*ik*_

#### (3) Plate model analysis

Plate model analysis refers to the clustering analysis to gather actors with similar characteristics in the plate into one plate, and the multiple actors as a whole to study their role and status in the spatial association network.

#### (4) The middleman role

The middleman role refers to a person in an intermediate position, who plays an intermediary role in the transmission of information in the same subgroup or in different subgroups. Through man-in-the-middle analysis, it is possible to clarify the position and role of each node in the information exchange within and between subgroups.

### 2.4 Data sources

In this study, there were 321 NEV industry listed companies on either the Shanghai or Shenzhen stock exchanges, representing all Chinese mainland provinces except for Xinjiang, Inner Mongolia, Guangxi, and Ningxia. Data on R&D personnel and R&D investment covering the 2013–2018 period were used, together with patent data (which shows a one year time lag) for 2014–2019. R&D personnel and R&D investment data were sourced from listed company annual reports, while the patent database was developed by searching the records of the State Intellectual Property Office of China.

For the super efficiency DEA model, in terms of input variables, the main dimensions of evaluating innovation investment are R&D personnel and R&D expenditures [23]. This article mainly includes listed company R&D personnel and R&D investment data for each province. R&D personnel can reflect the province’s investment in R&D personnel for innovation in the NEV industry. The R&D investment reflects the investment in the innovation of the NEV industry. As for output variables, it mainly includes the listed company patent data for each province. The patent data reflects the innovative achievements of the NEV industry. By comparing multiple indicators, scholars believe that patents are a suitable embodiment of innovation output [24,25]. The basic data for the spatial incidence matrix of influencing factors come from the China Statistical Yearbook, and from listed company annual reports. The innovation efficiency of provincial NEV listed enterprise replace as the innovation efficiency of the provincial NEV industry.

## Case study and discussion

### 3.1 Overall network structure characteristics and evolution trends

**Fig 1:**
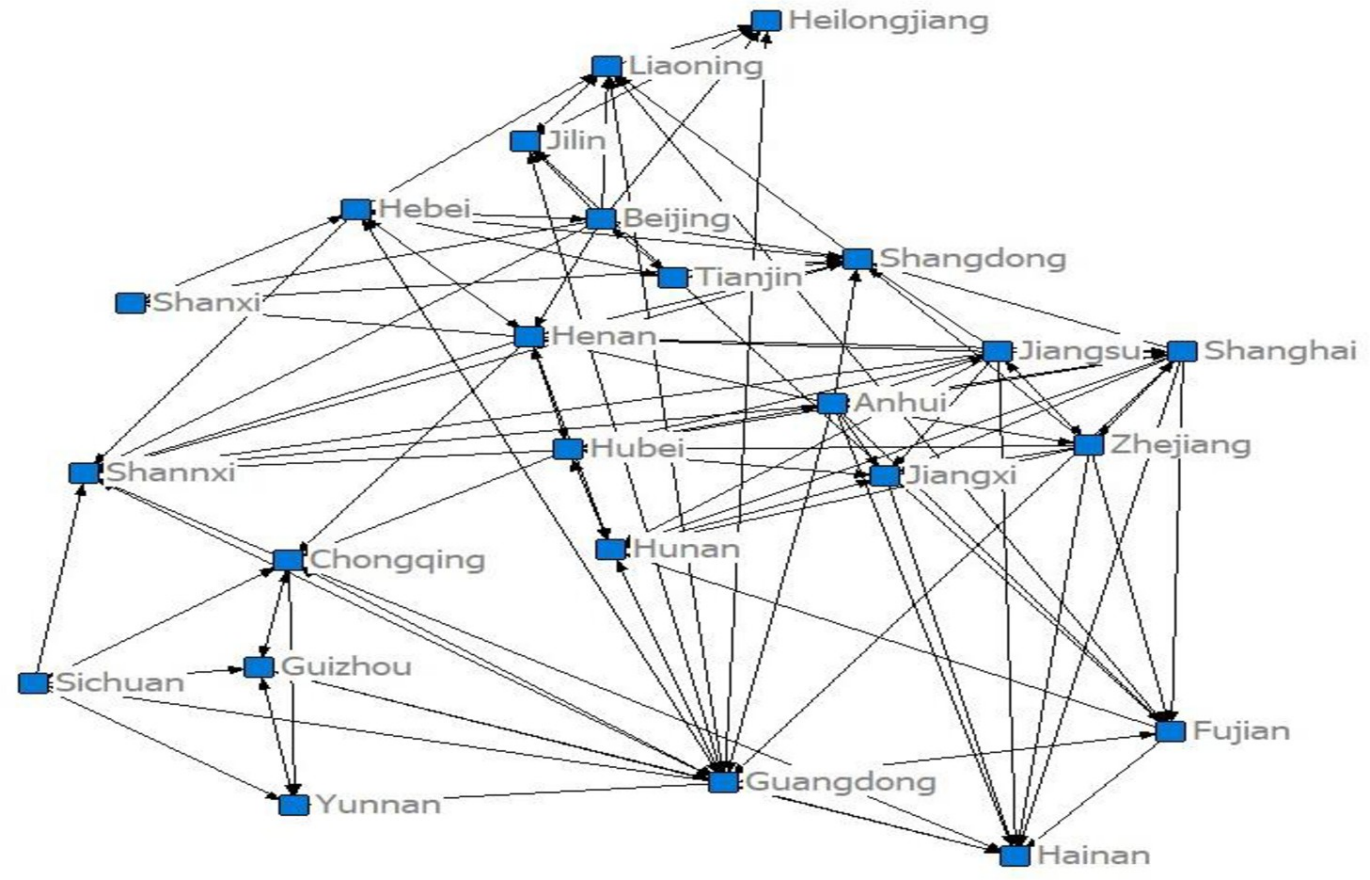
Spatial association network for innovation efficiency in China’s new energy vehicle industry in 2019.

In this study, we measured the innovation efficiency of the NEV industry from 2014–2019 using the super efficiency DEA model. The correlation matrix representing innovation efficiency between provinces, and covering the 2014–2019 period, was then obtained, using the modified gravity model. After binarization, the spatial correlation between provinces of innovation efficiency in the NEV industry was established, allowing the network diagram for 2019 as show in Fig 1 to be drawn.

Making the conclusion from Fig 1, in 2019 China’s NEV innovation efficiency spatial correlation structure network is composed of 24 provinces, the data of patent in Tibet, Gansu, Qinghai are zero.

#### (1) Network density and number of relationships

**Fig 2:**
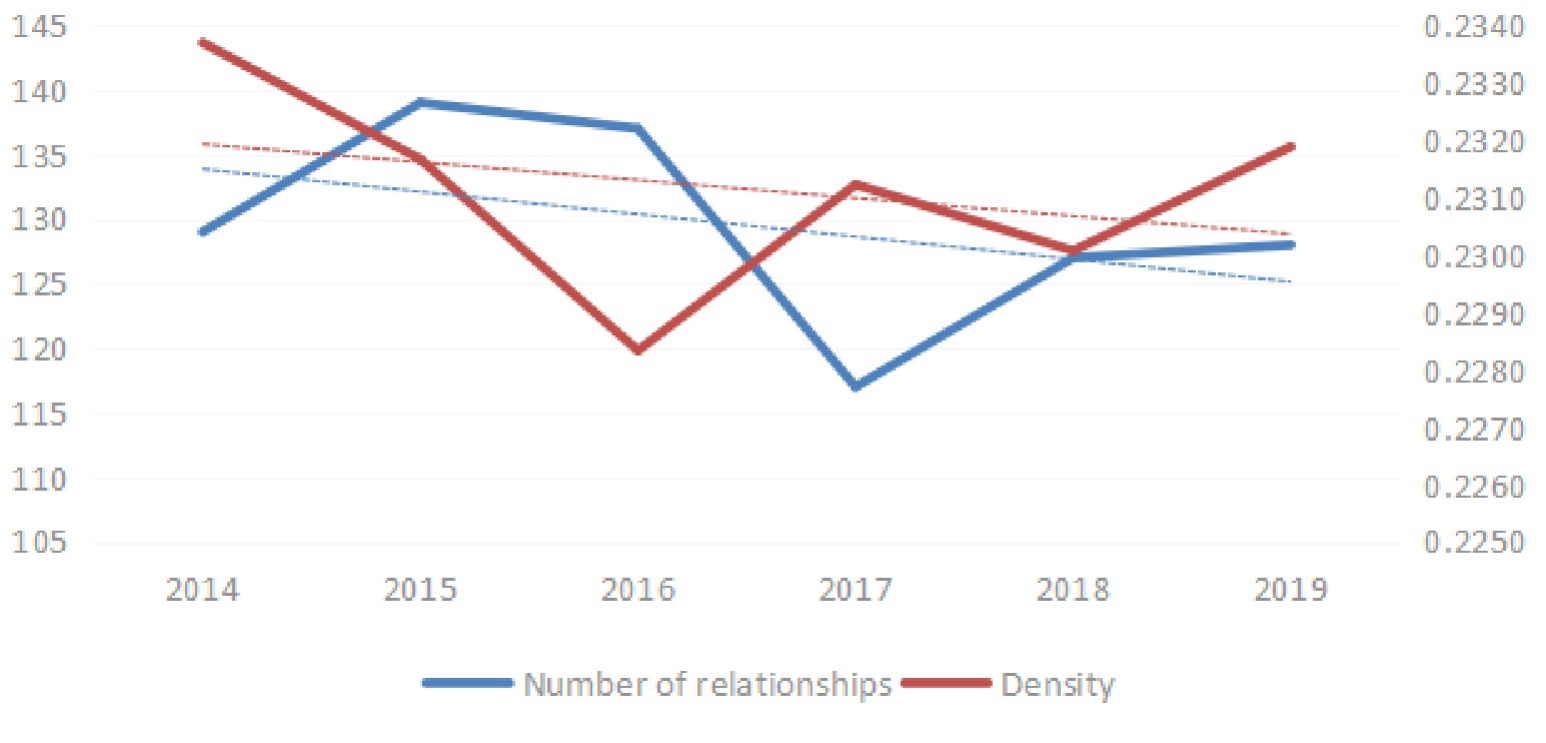
Trends in network density and number of relationships in China’s new energy vehicle industry.

Fig 2 shows the spatial correlation number and network density for innovation efficiency in the NEV industry among provinces from 2014–2019. In the Fig 2, network density and number of relationships in the SAN for innovation efficiency shows an overall downward trend, although this trend is not particularly large.

#### (2) Network hierarchy and network efficiency analysis

**Fig 3:**
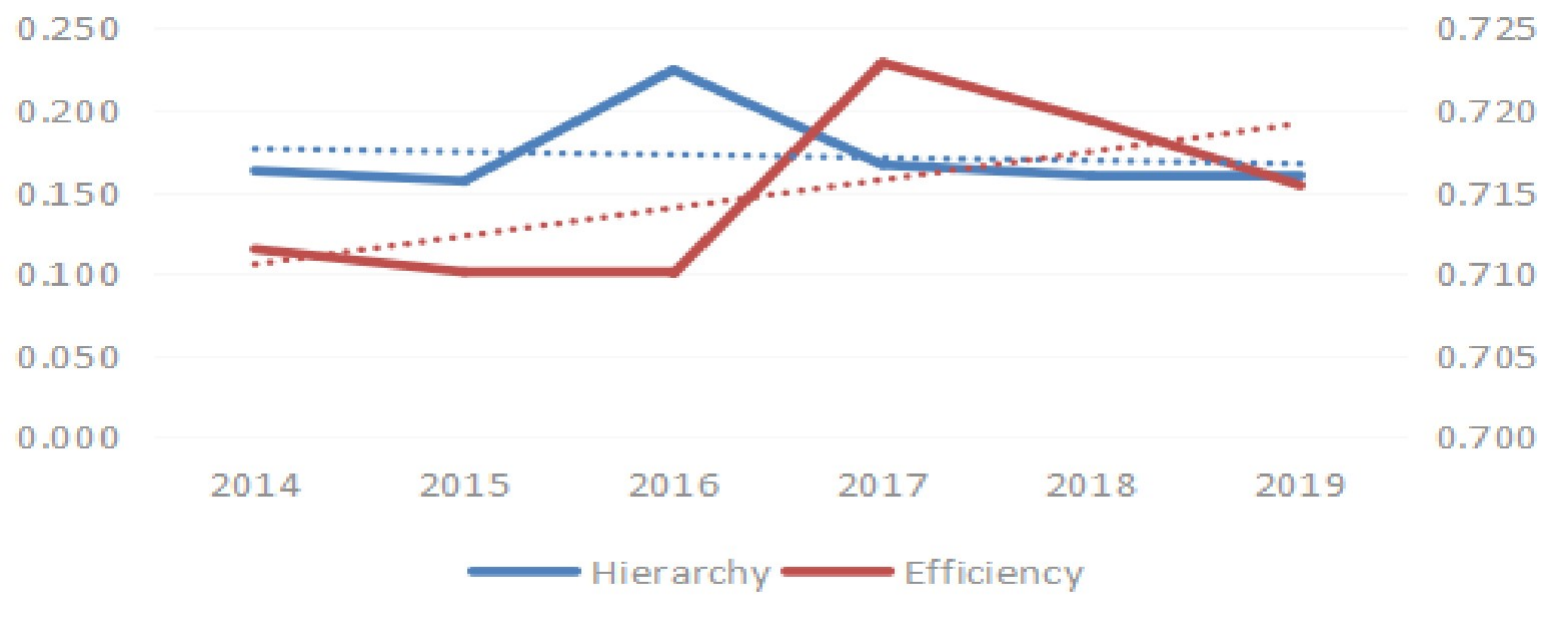
Trends in network hierarchy and network efficiency in China’s new energy vehicle industry.

Fig 3 shows the evolutionary trends in both the network hierarchy and network efficiency, covering 2014–2019, and it can be seen that the network hierarchy did not change significantly. The value for NEV industry network efficiency is much smaller than the maximum value of 1, reflecting the fact that there was no strict hierarchical structure in the NEV industry SAN. The network efficiency of the NEV industry was gradually improved, which showed that the stability of space network was affected.

### 3.2 Centrality analysis

This article analyzes the network characteristics of the innovation efficiency of NEV spatial network are by measuring the point, closeness, and betweenness centralities, to determine the status and role of each province and city in the innovation efficiency of NEV spatial correlation. Then, for studying the existence and level of influence exerted by particular regions in the SAN, we also measure the in-degree and out-degree from each province, with the results listed in Table 1. Patent data from Tibet, Qinghai, and Gansu are zero, so we delete these three locations from the SAN.

**Table 1:** Centrality analysis of innovation efficiency in the new energy vehicle spatial correlation network, for 2019.

Province	Point centrality				Closeness centrality		Betweenness centrality	
	Out-degree	In-degree	Degree	Rank	Closeness	Rank	Betweenness	Rank
Anhui	11	4	45.83	2	86.25	1	71.50	4
Beijing	9	3	37.50	8	69.27	10	9.57	13
Fujian	5	5	33.33	12	68.13	11	11.18	12
Guangdong	12	8	58.33	1	83.48	2	180.07	1
Guizhou	5	4	20.83	18	63.19	14	31.50	8
Hainan	0	9	37.50	9	40.11	24	0.00	19
Hebei	8	6	33.33	13	75.84	4	85.36	3
Henan	7	7	45.83	3	75.58	5	56.27	6
Heilongjiang	1	4	16.67	21	51.42	22	0.00	20
Hubei	6	7	37.50	10	75.32	6	107.70	2
Hunan	2	8	33.33	14	61.82	16	57.91	5
Jilin	3	5	20.83	19	61.92	15	23.76	10
Jiangsu	10	3	41.67	6	73.79	7	0.74	16
Jiangxi	3	6	29.17	16	58.71	17	1.46	15
Liaoning	4	6	29.17	17	63.61	13	12.65	11
Shandong	7	8	45.83	4	69.31	9	27.92	9
Shanxi	1	4	16.67	22	57.06	19	0.00	21
Shaanxi	0	10	41.67	7	42.50	23	0.00	22
Shanghai	11	3	45.83	5	83.41	3	3.67	14
Sichuan	4	3	20.83	20	53.83	20	0.67	17
Tianjin	3	3	12.50	24	58.27	18	0.00	23
Yunnan	1	4	16.67	23	52.61	21	0.00	24
Zhejiang	9	3	37.50	11	73.79	8	0.60	18
Chongqing	6	5	33.33	15	65.79	12	33.48	7
Mean	5	5	32.99	—	65.21	—	29.83	—

#### (1) Point centrality

Point centrality is used to reflect the status of each province in the SAN. The average values for in-degree, out-degree, and degree centrality for the Chinese provinces were 5, 5, and 32.99, respectively. The centrality degrees for 15 provinces—Anhui, Beijing, Fujian, Guangdong, Hainan, Hebei, Henan, Hubei, Hunan, Jiangsu, Shandong, Shaanxi, Shanghai, Zhejiang, and Chongqing—exceeded the average. Of these, Guangdong, Anhui, Henan, Shandong, and Shanghai made up the top five, occupying central positions, and having more connections with the other provinces.

The average out-degree value was 5, with 11 provinces—Anhui, Beijing, Guangdong, Hebei, Henan, Hubei, Jiangsu, Shandong, Shanghai, Zhejiang, and Chongqing—exceeding this. Guangdong, Anhui, Shanghai, Jiangsu, Beijing, and Zhejiang made up the top six, with values approximately twice that of the average, indicating that these six provinces have spilled their NEV industry innovation efficiency over to other provinces.

The average in-degree value was also 5, with nine provinces—Guangdong, Hainan, Henan, Hubei, Hunan, Jiangxi, Liaoning, Shandong, and Shaanxi—exceeding this. Shaanxi, Hainan, Guangdong, Hunan, and Shandong had the highest in-degree results, with values approximately twice the average—showing that innovation efficiency had spilled over from other provinces into these five.

#### (2) Closeness centrality

Closeness centrality is a key indicator for judging the difficulty of association between provinces in the SAN. The results listed in Table 1 showed that the average closeness centrality value for the provinces in China was 65.21. There being12 provinces—Anhui, Beijing, Fujian, Guangdong, Hebei, Henan, Hubei, Jiangsu, Shandong, Shanghai, Zhejiang, and Chongqing—exceed the average. Of these, Anhui, Guangdong, Shanghai, Henan, and Hebei had the highest values, indicating that they enjoyed superior locations, being spatially close to several other provinces, and were therefore more likely to have spatial associations with other provinces and cities in the SAN.

#### (3) Betweenness centrality

Betweenness centrality is used to measure the abilities of provinces to control resources in the SAN. The betweenness centrality average among the provinces was 29.83. Values calculated for Anhui, Guangdong, Guizhou, Hebei, Henan, Hubei, Hunan, and Chongqing provinces exceeding this average, revealed that these eight performed "bridge" roles in the network, with strong control over its form, and acted as key network resourcing points.

### 3.3 Plate model analysis

By applying a block model, then we proceeded to conduct a more in-depth analysis of spatial correlations between NEV innovation efficiency nodes, which reveals the spatial clustering, relationships, and overflow paths in the network. The research uses the CONCOR method to divide the NEV innovation efficiency network into four different plates (see Table 2). Anhui, Jiangxi, Fujian, Shanghai, Zhejiang, and Jiangsu provinces constituted the first plate, with Hainan, Henan, Shandong, Hunan, and Hubei making up the second. There were eight members in the third plate—Heilongjiang, Jilin, Liaoning, Beijing, Hebei, Shaanxi, Shanxi, and Tianjin—and five in the fourth plate, namely Guangdong, Sichuan, Yunnan, Guizhou, and Chongqing.

**Table 2:** Innovation efficiency spillover effects within the Chinese new energy vehicle industry correlation plates in 2019.

Plate	Contacts received		Contacts sent		Expected internal relationship	Actual internal relationship	Plate properties
	Inside	Outside	Inside	Outside			
First plate	19.00	5.00	19.00	30.00	0.22	0.39	Net spillover
Sceond plate	7.00	32.00	7.00	15.00	0.17	0.32	Net beneficial
Third plate	20.00	21.00	20.00	9.00	0.30	0.69	Bidirectional spillover
Forth plate	16.00	8.00	16.00	12.00	0.17	0.57	Brokers

The location and attributes of each plate in the NEV industry innovation efficiency SAN are shown in Table 2. As can be seen in Table 2, there are 128 correlations in the network, of which 76 (59.4%) are within the plates, and 52 (40.6%) are among the plates, showing that NEV industry innovation efficiency spatial spillover was mainly of the internal overflow.

There are 24 internal relations in the first plate, five receiving spillover relations from the other plates, and 30 examples of contacts being sent to the other plates. The expected internal relationship ratio was therefore 0.22, while the actual internal proportion was 0.39, indicating that the first plate was a net spillover plate. There are seven relationships within the second plate, 32 relationships that accepted spillovers from other plates, and 15 relationships that spilled over to other plates. The expected internal proportion is 0.17, while the actual figure is 0.32, indicating that the second plate was a net beneficial plate. There are 20 relationships within the third plate, 21 relationships that accepted spillovers from others, and nine relationships that spilled over to others plates. The expected internal proportion was 0.30, while the actual internal proportion was 0.69, indicating that the third plate had spillover effects with both itself and other plates, in an outcome we have referred to as the third plate bidirectional spillover. The fourth plate had eight relationships that accepted spillovers from other plates, and 12 relationships that spilled over to others. The expected internal proportion was 0.17, while the actual internal value was 0.57, indicating that the fourth plate not only had spillover effects to other sectors, but also accepted spillover relationships from other plate.

To further study the spatial correlations between the innovation efficiency of each plates, the density matrix of each plate was calculated. In 2019, the network density for spatial correlations across the whole network was 0.232. If the individual plate network density is > 0.232, the network density for those plates are greater than that of the whole network, indicating that innovation efficiency would be more concentrated there, we use 1 to represent the value, otherwise, the value is zero.

As the results presented in Table 3 showed that the second plate had a relationship with the first plate, while the fourth plate did not have a relationship with any other plate. We noted that the fourth plate was located in Southwest China, and have since hypothesized that connections between the fourth plate and the others were weak because of the underdeveloped nature of its transportation infrastructure.

**Table 3:** Density matrix and image matrix of innovation efficiency of China’s the NEV industry in 2019.

Plate	Density matrix				Image matrix			
	First plate	Sceond plate	Third plate	Forth plate	First plate	Sceond plate	Third plate	Forth plate
First plate	0.633	0.767	0.083	0.100	1	1	0	0
Sceond plate	0.1	0.35	0.25	0.080	0	1	1	0
Third plate	0.021	0.125	0.357	0.075	0	0	1	0
Forth plate	0.033	0.16	0.175	0.800	0	0	0	1

### 3.4 The middleman role

This paper also makes a further in-depth analysis of the role played by each province in the four plates through the role of middle-man. The statistics between the provinces on how often they each acted as different types of middleman differed, it could be seen from the data listed in Table 4 that there being five provinces play no roles with the middleman, most of them are the remote area of China Mainland. The data presented in Table 4 showed that the 19 provinces played different middleman roles in the different plates. For example, in the first plate, Anhui and Fujian acted as agents, playing very important roles communicating with other plates. Anhui also acted as the main gatekeeper for the first plate, with other plates interacting with provinces in the first plate mainly through Anhui Province. Henan and Shandong in the second plate were found to be acting as major liaison centers, having a significant impact on NEV industry innovation efficiency connections between the other two plates. Henan also acted as the major gatekeeper in the second plate, indicating that the other plates connected with the provinces within the second plate largely through Henan Province. Hubei was found to be acting as a major representative, indicating that second plate provinces all communicated with each other through Hubei Province. In the third plate, we saw that Hebei fulfilled the roles of main coordinator, gatekeeper, agent, and contact middleman simultaneously, making this province the most important for internal and external exchanges in the third plate, playing a pivotal role there. To expand on this a little, Hebei was acting not only as coordinator for NEV industry innovation efficiency of spatial correlation between the provinces and cities within the third plate, but was also the main gatekeeper for other plates seeking to influence third plate provinces. Hebei Province played a representative role, indicating that third plate cities and provinces communicated with the other plates through Hebei. Hebei fulfilled an important liaison role for communication between other plates, while Beijing also acted as a major coordinator in the third plate. In the fourth plate, Guangzhou fulfilled the roles of main gatekeeper, representative, consultant, and liaison middleman. Guizhou acted as a major coordinator, playing an important role in communicating with the provinces within the fourth plate.

**Table 4:** Number of middlemen in provinces in the innovation efficiency correlation network of China’s new energy vehicle industry.

Plate	Province	Coordinator	Gatekeeper	Rep.	Consultant	Liaison	Total
One	Anhui	1	4	5	2	1	13
	Jiangxi	0	0	1	2	0	3
	Fujian	0	0	8	0	2	10
	Shanghai	1	0	4	0	0	5
	Zhejiang	1	0	1	0	0	2
	Jiangsu	0	0	4	0	0	4
Two	Hainan	0	0	0	0	0	0
	Henan	3	5	3	0	12	23
	Shandong	0	2	3	3	22	30
	Hunan	0	2	1	1	1	5
	Hubei	1	3	5	1	8	18
Three	Heilongjiang	0	0	0	0	0	0
	Jilin	1	1	2	0	0	4
	Beijing	7	2	1	0	0	10
	Liaoning	2	3	4	0	2	11
	Hebei	7	6	6	0	4	23
	Shanxi	0	0	0	0	0	0
	Shaanxi	0	0	0	0	0	0
	Tianjin	0	0	0	0	0	0
Four	Guangdong	0	24	13	6	21	64
	Sichuan	0	0	1	0	0	1
	Yunnan	0	0	0	0	0	0
	Guizhou	4	0	2	0	0	6
	Chongqing	1	8	2	2	0	13

Up to this point, we have analyzed change trends in NEV industry SAN innovation efficiency through various regions, together with the overall structure, individual network characteristics, and the interrelationships of the study regions. Questions relating to how the spatial relationships of NEV industry innovation efficiency were formed, and on the phenomena which either promoted or hindered generation of innovation efficiency spatial associations remained unanswered, however. To address this, we have included analysis of factors influencing spatial correlations associated with NEV industry innovation efficiency in the following text. The aim here has been to provide new ideas on how to coordinate development of NEV industry innovation efficiency.

## 4. Analysis of factors influencing spatial correlations in NEV industry innovation efficiency

In this study, QAP analysis was used to investigate formation mechanisms associated with the spatial correlations and spillover pathways affecting NEV industry innovation efficiency. Influencing factors were reviewed, with the intention of being able to provide theoretical guidance for adjusting NEV industry development policies in different regions, in line with local conditions.

### 4.1 Influencing factor selection and model establishment

Some researchers found that spatial correlation strengths in innovation and development were closely related to geographic separation [26–28], with innovation and development spillovers found to be more prevalent between neighboring provinces. Innovation efficiency spatial correlations may be related to differences in regional economic development. According to Chen et al. [29], factors such as enterprise size and the strength of government support in different regions also affected such spatial correlations. These were therefore regarded as the factors influencing spatial network development. To the extent that differences in R&D personnel and capital investment were also considered to be factors influencing spatial correlations and were seen as being connected to investment in R&D personnel and to allocation of R&D capital. Thus, the following model was constructed:

F=(D,N,S,T,R,F,P) (10)

where *F* represents the spatial correlation matrix of NEV industry innovation efficiency in 2019, *D* represents the matrix of spatial adjacency relationships, and *N* represents the difference matrix for regional economic development. *S* represents the enterprise scale difference matrix (defined as the ratio of total assets of NEV listed enterprises in each province, to the number of listed enterprises), while *T* represents the difference matrix of government support (measured by the proportion of government subsidies in the total assets of enterprises). *R* represents the difference matrix of talent investment (measured as the number of full-time equivalent R&D personnel), with *F* representing the difference matrix of R&D investment, and *P* representing the difference matrix of the intellectual property protection index.

These variables were adopted as relational data, which is to say that the observed values were not assumed to be independent of each other, and that there may be high levels of correlation between the variables. This meant that the traditional, ordinary least squares method was not suitable for parameter estimation, with the non-parametric QAP method used instead to analyze spatial correlations between regional innovation and development. Since QAP did not require assumption that independent variables were uncorrelated, the "multicollinearity" of driving factors did not need to be considered, making it more suitable for hypothesis testing based on relational data.

### 4.2 Analysis of influencing factor with QAP

In this study, QAP was carried out using UCINET software, achieving an R^2^ of 0.243 after completing calculations and adjustments. Model interpretation, in terms of variation of the dependent variable “innovation efficiency association network” was 24.3%—which was significant at the 1% level. According to the QAP results listed in Table 5, spatial adjacency was significant at the 1% level, which suggested that geographic proximity had a significant impact on innovation efficiency spatial correlations—that is, the closer locations were, the more likely they were to have significant spatial correlations. The difference of R&D investment was significant at the 5% level, indicating that such differences could affect spatial correlations in NEV industry innovation efficiencies. The other influencing factors were significant at > 10%, showing that their effects on innovation efficiency spatial correlations were not significant.

**Table 5:** Quantitative assignment procedure regression analysis results.

Variable matrix	Un-standardized Independent	Standardized Coefficient	Sig.	P ≥ 0	P ≤ 0
Intercept	0.15	0.00	—	—	—
S	- 0.04	- 0.05	0.15	0.85	0.15
D	0.58	0.50	0.00	0.00	1.00
T	- 0.04	- 0.04	0.22	0.79	0.22
N	- 0.05	- 0.05	0.14	0.86	0.14
F	0.09	0.11	0.05	0.05	0.95
P	0.04	0.05	0.17	0.17	0.83
R	- 0.03	- 0.04	0.23	0.77	0.23

## 5 Conclusions and suggestions

### 5.1 Conclusions

After using the super-efficiency DEA model to calculate the innovation efficiency of NEV in the province, we use UCINET software to construct and analyze the spatially relevant network structure of innovation efficiency in China’s NEV industry, and draw some conclusions.

Firstly, the innovation efficiency of China’s NEV industry presents the significant and complex spatial correlation network structure, There is a close spatial correlation between the innovation efficiency of the NEV industry and there is no strict hierarchical structure in the spatial correlation network of innovation efficiency of NEV industry.

Secondly, the role of each region in the spatial correlation network of the innovation efficiency of the NEV industry is different. The current literature studies the NEV industry from the perspective of regional innovation efficiency differences, and regards the regional innovation system as an independent entity [11,12]. This article mainly analyzes the spatial correlation network of the innovation efficiency of the NEV industry from the three aspects of centrality, plate model and middleman, which expands the research content of NEV innovation to a certain extent.

From the perspective of the centrality, we found that Guangdong, Anhui, Henan, Shandong, Shanghai, and other eastern and central provinces occupied key network positions and played vital roles. Of these, Anhui, Guangdong, Guizhou, Hebei, Henan, Hubei, Hunan, and Chongqing fulfilled clear "intermediary" and "bridge" roles.

From the perspective of the plate mode, the provinces in the network can be divided into four plates, with the first plate consisting of Anhui, Jiangxi, Fujian, Shanghai, Zhejiang, and Jiangsu provinces, which played "net spillover" roles. The five members of the second plate—Hainan, Henan, Shandong, Hunan, and Hubei—constituted a “net beneficial plate,” in that they benefitted from spillovers from other provinces. Heilongjiang, Jilin, Liaoning, Beijing, Hebei, Shaanxi, Shanxi, and Tianjin provinces made up the third plate, which was found to be a “bidirectional spillover plate,” while Guangdong, Sichuan, Yunnan, Guizhou, and Chongqing constituted the fourth plate, fulfilling the "brokers plate" role.

From the perspective of the middleman, the central and eastern regions act as intermediaries frequently in spatial association networks, which plays an intermediary role in establishing relationships within and between sectors.

Thirdly, it was found that R&D investment levels and inter-provincial proximities also had significant positive effects on the spatial correlations found for the NEV innovation efficiency. The regional economic development, the enterprise scale, government support, the talent investment, the intellectual property protection index have no significant on the spatial correlation effect on the innovation efficiency of the NEV industry.

### 5.2 Policy implications

On the basis of understanding the structural characteristics of the spatial correlation network of the innovation efficiency of the NEV industry, this spatial correlation is used as a suggestion for the formulation of NEV development policies in various regions. Then, the regions actively cooperate with other regions to promote the collaborative and innovative development of China’s NEV industry. While maintaining the technological development of NEVs, the provinces in the center of the SAN should guide technological development in other plate provinces. They should also actively cooperate with them in space-related networks, in order to ensure that full use is made of the innovative resources available in these other regions. For the “intermediary” and “bridge” areas in the spatial association network, while improving its own technological innovation capabilities, it is necessary to spread technology to a larger spatial scope

Establishment of a collaborative innovation mechanism for the NEV industry should be accelerated. Provinces in the "two-way spillover" and "net income" sectors should take advantage of the central government policy framework, to encourage NEV industry development. They should also be vigorous in their pursuit of technical innovation, and in developing their own NEV industry, based on their advantages and characteristics. Provinces in the "net spillover" and "broker" sectors should formulate preferential policies to attract the NEV industry into their provinces. They should welcome existing NEV industry participants from more developed regions into their region, together with their advanced science and technology, while continuing to transform and upgrade their own NEV industry elements.

The dual roles of market regulation and government macro-control need to be fully developed and coordinated. On the one hand, the government should actively take measures to increase support for the NEV industry through "tangible hands" policies, so as to improve China’s NEV industry innovation efficiency overall. On the other hand, We should reduce the administrative intervention of the government, and strength the competition and cooperation among enterprises in the NEV industry by using the market mechanism of competition, cooperation, supply and demand.

The research of this article still has the limitation. This article only analyzes the factors that affect the spatial correlation of the innovation efficiency of the high-tech energy automobile industry, but fails to further analyze the degree of influence of these factors and the mechanism on the spatial correlation network. About the limitation, we will do further explore in future research.

## Supporting information

S1 Dataset (XLS)
